# Clinical benefit of paclitaxel/carboplatin plus bevacizumab with zoledronic acid in pulmonary epithelioid hemangioendothelioma complicated by hypertrophic pulmonary osteoarthropathy and cardiac tamponade: a case report

**DOI:** 10.3389/fonc.2026.1855346

**Published:** 2026-06-02

**Authors:** Saki Tsuchimochi, Hiromichi Nakajima, Yukako Hattori, Shioto Oda, Hiroki Imada, Tetsuro Taki, Anna Matsuoka, Shogo Watanabe, Akira Hirota, Mai Shimura, Misao Fukuda, Chikako Funasaka, Kenichi Harano, Nobuaki Matsubara, Yoichi Naito, Ako Hosono, Genichiro Ishii, Toru Mukohara

**Affiliations:** 1 Department of Medical Oncology, National Cancer Center Hospital East, Kashiwa, Japan; 2 Department of Experimental Therapeutics, National Cancer Center Hospital East, Kashiwa, Japan; 3 Department of General Internal Medicine, National Cancer Center Hospital East, Kashiwa, Japan; 4 Department of Palliative Medicine, National Cancer Center Hospital East, Kashiwa, Japan; 5 Department of Diagnostic Radiology, National Cancer Center Hospital East, Kashiwa, Japan; 6 Department of Pathology and Clinical Laboratories, National Cancer Center Hospital East, Kashiwa, Japan; 7 Department of Pediatric Oncology, National Cancer Center Hospital East, Kashiwa, Japan

**Keywords:** bevacizumab, Cardiac tamponade, epithelioid hemangioendothelioma, hypertrophic pulmonary osteoarthropathy, pericardial effusion, pleural effusion, pulmonary epithelioid hemangioendothelioma, zoledronic acid

## Abstract

Epithelioid hemangioendothelioma (EHE) is a rare vascular neoplasm lacking a standard systemic therapy. Pulmonary EHE (PEH) may be aggressive if accompanied by serosal effusion and has occasionally been reported in association with hypertrophic pulmonary osteoarthropathy (HPOA). Here, we report a case of a 33-year-old woman who had never smoked and presented with chronic cough, arthralgia, and digital clubbing. Imaging revealed a left upper lobe mass with mediastinal invasion, lymphadenopathy, and pericardial effusion. The patient developed cardiac tamponade that required emergent pericardial drainage. Histopathology from the transbronchial biopsy and mediastinal lymph node sampling showed an epithelioid endothelial tumor positive for CD31, D2-40, factor VIII, and ERG with nuclear CAMTA1 expression, confirming EHE. Bone scintigraphy and ankle magnetic resonance imaging revealed symmetric periosteal-predominant abnormalities consistent with HPOA. Zoledronic acid was administered for HPOA, and paclitaxel/carboplatin plus bevacizumab was initiated with palliative intent. Follow-up imaging during treatment showed marked reduction of the pericardial and pleural effusions, with improvement in arthralgia and activities of daily living. Although the benefit was transient, this case suggests that systemic chemotherapy in combination with bevacizumab may have contributed not only to temporary effusion control in advanced PEH but also to relief of PEH-associated HPOA, while concomitant zoledronic acid may also have supported symptom improvement.

## 1 Introduction

Epithelioid hemangioendothelioma (EHE) is an extremely rare vascular endothelial tumor with an incidence of <1 case per 1,000,000 person-years. Its clinical course is highly heterogeneous, ranging from indolent to rapidly progressive. Reportedly, >50% of patients present with multifocal involvement across multiple organs upon diagnosis (1, 2). At the molecular level, approximately 90% and 10% of EHE harbor the *WWTR1*–*CAMTA1* and *YAP1*–*TFE3* fusions, respectively; these fusions are considered specific biomarkers of EHE, regardless of the site of origin (3).

Pulmonary EHE (PEH) accounts for approximately 20% of all EHE cases. Because the clinical symptoms and radiological findings are nonspecific, PEH is often misdiagnosed as an infectious disease or organizing pneumonia. Considering its endothelial origin, EHE exhibits a high expression of vascular endothelial growth factor (VEGF), contributing to the development of pleural effusion, pericardial effusion, and disease progression (4). Accordingly, PEH accompanied by pleural and/or pericardial effusion is associated with a poor prognosis, and if surgical resection or definitive radiotherapy is not feasible, systemic therapy is the mainstay of treatment. However, no standard systemic regimen with established efficacy exists (1).

PEH can be complicated by hypertrophic pulmonary osteoarthropathy (HPOA), which is characterized by digital clubbing, arthralgia, and periosteal reaction. HPOA is a well-recognized paraneoplastic syndrome, and approximately 90% of secondary HOA cases are associated with malignancies, most commonly non-small cell lung cancer (5). Although uncommon, several cases of EHE associated with HPOA have been reported (6–11), suggesting that HPOA may be underrecognized. The proposed mechanisms include excessive VEGF production and platelet–endothelial interactions, but the pathophysiology remains unclear (12). Management of secondary HPOA depends on controlling the underlying disease. In refractory cases, bisphosphonates have been reported to be effective in improving pain and arthritis (13); however, their clinical significance in HPOA associated with PEH is unclear.

Here, we report a case of advanced PEH in a 33-year-old woman with HPOA and cardiac tamponade, in whom paclitaxel/carboplatin plus bevacizumab (TC+Bev) combined with zoledronic acid was associated with clinically meaningful benefits. Our experience from this case provides valuable clinical insights into the potential use of anti-VEGF therapy in PEH with a VEGF-driven pathophysiology and highlights the benefits of concomitant bisphosphonate therapy for PEH-associated HPOA.

## 2 Case description

**Figure 1:**
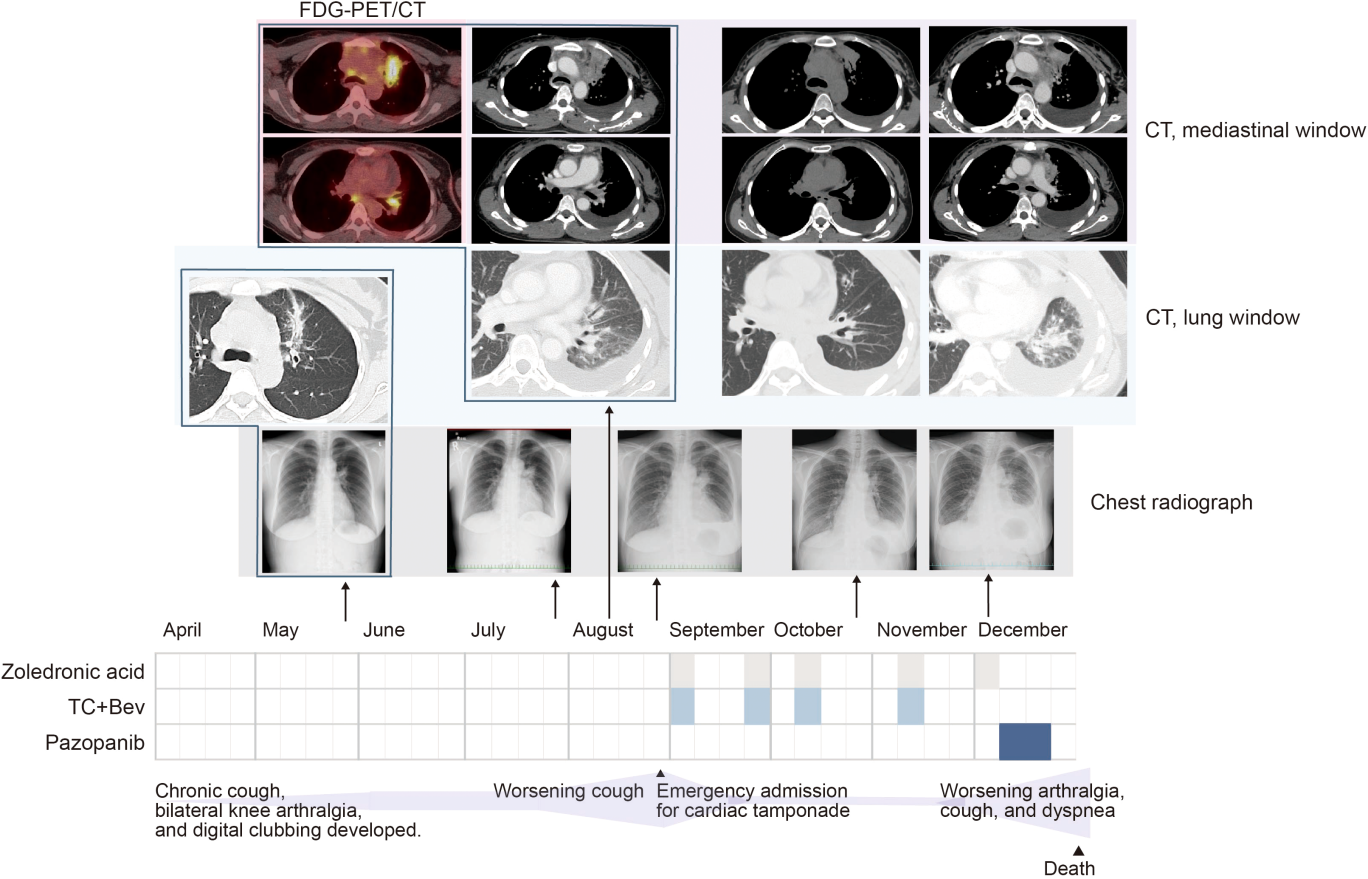
Timeline of clinical course and serial thoracic imaging. The upper two rows show FDG-PET/CT and CT images in the mediastinal window, with the leftmost panel representing FDG-PET/CT and the remaining panels representing CT. The third row shows CT images in the lung window, and the bottom row shows serial chest radiographs. In May 20XX, initial CT revealed an infiltrative lesion in the left upper lobe. In August 20XX, FDG-PET/CT demonstrated abnormal uptake in a lesion extending from the left upper lobe into the mediastinum, and concurrent CT showed an atelectasis-like mass along the mediastinal aspect. Chest radiography at the time of cardiac tamponade is also shown. In October 20XX, post-chemotherapy CT showed marked resolution of pericardial and pleural effusions. In December 20XX, follow-up CT demonstrated disease progression, with enlargement of the left upper lobe lesion, increased pleural effusion causing near-complete opacification of the left lung, and new reticular opacities with interlobular septal thickening in the right lower lobe, consistent with lymphangitic carcinomatosis. After three cycles of TC+Bev with zoledronic acid, radiographic regression was accompanied by relief of arthralgia and improvement in ECOG PS from 2 to 1. In contrast, radiographic progression from late November to early December coincided with recurrence of arthralgia, worsening dyspnea and cough, and subsequent deterioration in ECOG PS to 3.

The clinical course is summarized in a timeline (**Figure 1**). A 33-year-old woman who had never smoked presented with chronic cough and musculoskeletal symptoms. Her medical history included bronchial asthma and left hemithyroidectomy for papillary thyroid carcinoma at 22 years of age. Her older sister had died of leukemia in childhood, but no other family history suggestive of a hereditary cancer syndrome was identified. Her obstetric history was gravida 2, para 2. She lived with her family and had been independent in activities of daily living before symptom onset. No psychosocial factor that materially interfered with the diagnostic work-up or treatment delivery was identified. She had no relevant occupational or environmental exposures. No germline genetic testing was performed. In April 20XX, she developed chronic cough, bilateral knee arthralgia, and digital clubbing. In May 20XX, she visited a local hospital in which chest computed tomography (CT) revealed an infiltrative lesion in the left upper lobe, and post-viral organizing pneumonia was suspected. She was evaluated by a rheumatologist, and because rheumatoid factor, anti-cyclic citrullinated peptide antibody, and other autoantibodies were negative, her arthralgia was provisionally attributed to post-infectious reactive arthritis. She was therefore followed with nonsteroidal anti-inflammatory drugs (NSAIDs), prednisolone (10 mg), and sulfasalazine (1g). Autoimmune arthritides, including rheumatoid arthritis and seronegative inflammatory arthritis, were considered less likely because inflammatory markers, including C-reactive protein and erythrocyte sedimentation rate, were not elevated. Digital clubbing was also noted, although it was not fully explained by this provisional diagnosis. Although her arthritic symptoms improved, the cough worsened, and she revisited the hospital in August 20XX. Repeat CT revealed a left upper lobe mass with mediastinal lymphadenopathy and pericardial effusion. 18F-fluorodeoxyglucose positron emission tomography/computed tomography (FDG-PET/CT) revealed abnormal FDG uptake corresponding to a lesion extending from the left upper lobe to the mediastinum. Bronchoscopic biopsy specimens were obtained from the left B3c bronchus. Additionally, endobronchial ultrasound-guided needle aspiration and intranodal forceps biopsy were performed on the right lower paratracheal and subcarinal lymph nodes (IASLC lymph node map).

**Figure 2:**
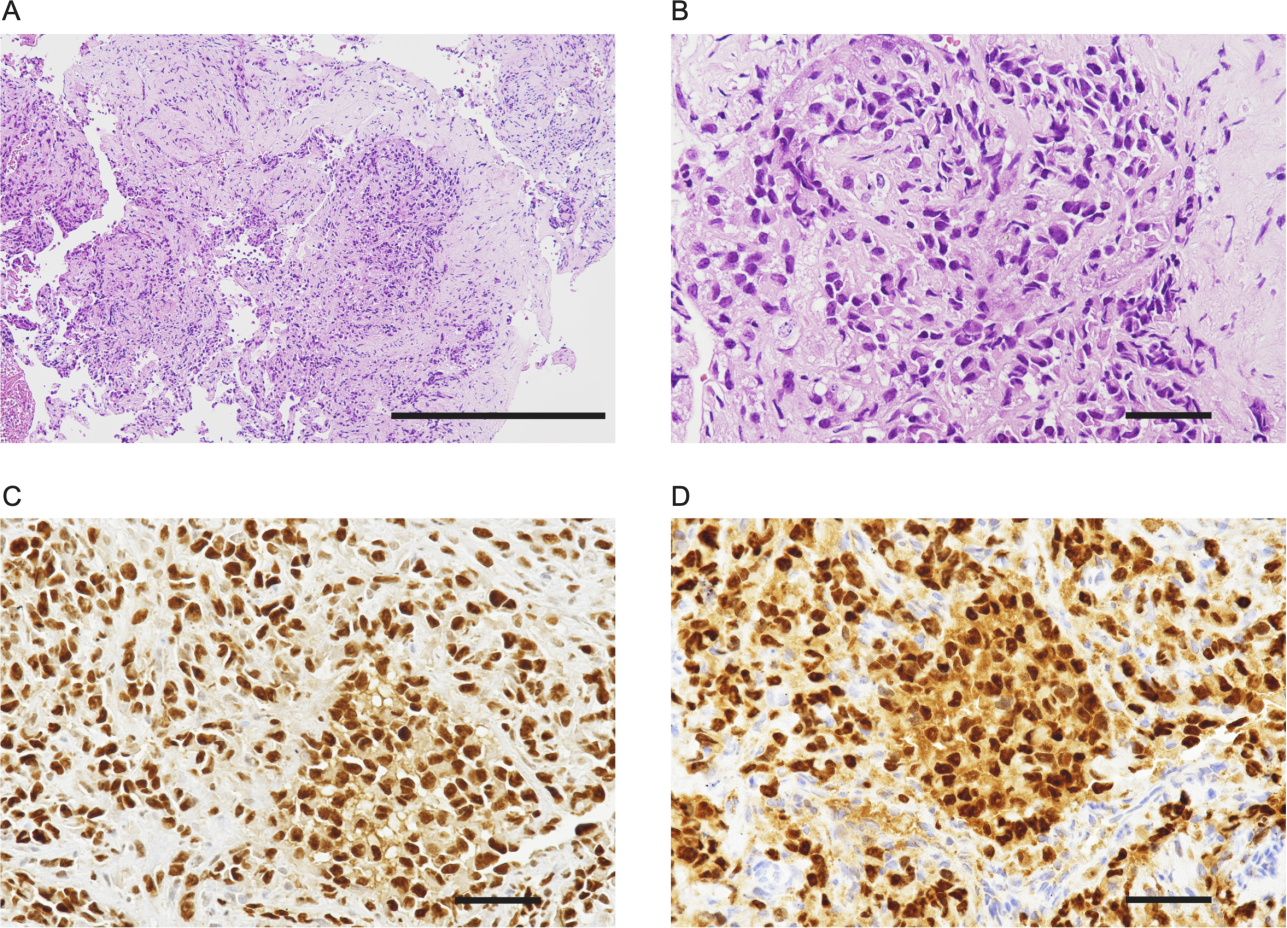
Histopathological findings of pulmonary epithelioid hemangioendothelioma. **(A)** Hematoxylin and eosin (H&E) staining at low magnification shows an epithelioid neoplasm with an infiltrative growth pattern invading the surrounding lung parenchyma. **(B)** H&E at higher magnification demonstrates tumor cells with eosinophilic cytoplasm and enlarged nuclei arranged in cords to small nests, characteristic of EHE. **(C)** Immunohistochemistry for ERG (an endothelial-specific transcription factor) shows nuclear positivity, confirming endothelial differentiation. **(D)** Immunohistochemistry for CAMTA1 shows nuclear positivity, supporting *WWTR1–CAMTA1* fusion-associated EHE. Scale bars: **(A)**, 500 μm; **(B–D)**, 50 μm.

Histopathological examination revealed tumor cells with eosinophilic cytoplasm and enlarged nuclei proliferating in cords to small nests (**Figures 2A, B**). Immunohistochemistry showed positivity for CD31, D2-40, factor VIII, and ERG, whereas CD34 was negative. ERG and CAMTA1 immunostaining are shown in **Figures 2C, D**. The Ki-67 labeling index was 20–30%. Molecular testing for *WWTR1*–*CAMTA1* or *YAP1*–*TFE3* fusions was not performed; however, the diagnosis of EHE was established based on characteristic histopathological and immunohistochemical findings, including nuclear CAMTA1 expression.

**Figure 3:**
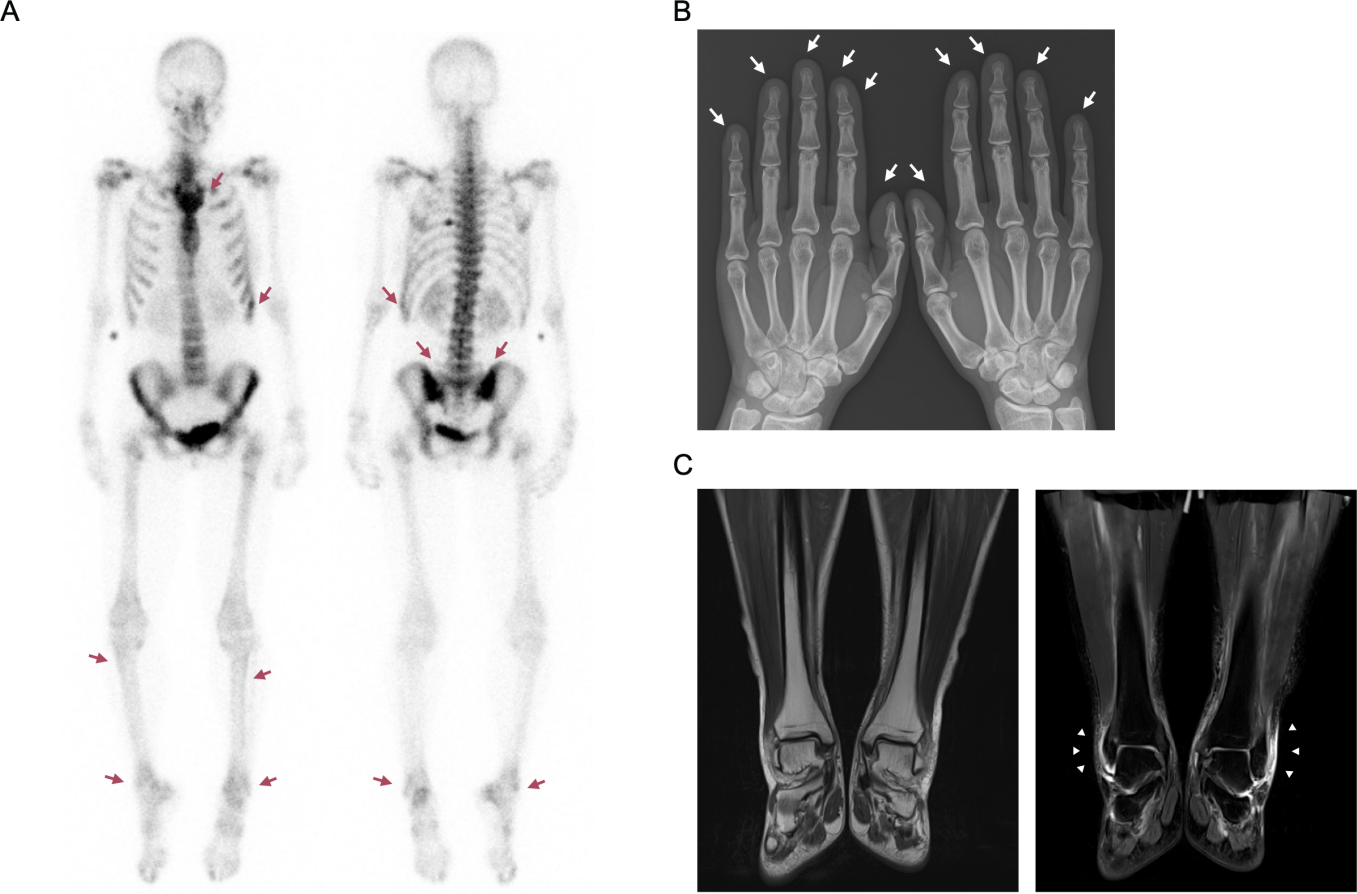
Imaging findings supporting the diagnosis of hypertrophic pulmonary osteoarthropathy (HPOA). **(A)** Whole-body bone scintigraphy with anterior view (left) and posterior view (right). Arrows indicate areas of radiotracer uptake. The images demonstrate diffuse, symmetric, periosteal-predominant radiotracer uptake consistent with HPOA, with prominent uptake in the sacroiliac joints and sternum, and additional uptake in the ribs, tibial cortex, and ankles. **(B)** Hand radiograph shows increased soft-tissue shadow compatible with digital clubbing. Arrows indicate the swelling of the digits. **(C)** Ankle MRI using T2-weighted Three-point Dixon images (TR/TE = 3310/70 ms, FA = 120°). Both in-phase and water-only images are shown. No abnormality is identified on the in-phase image (left), while the water-only image (right) demonstrates high signal intensity in the periosteum of the ankle joint, as indicated by arrowheads.

The patient was referred to our hospital in late August 20XX. Three days after referral to our hospital, she developed acute worsening of dyspnea. Her blood pressure was 119/82 mmHg, heart rate 120 beats/min, body temperature 37.0 C, and oxygen saturation 92% on room air. Echocardiography demonstrated pericardial effusion and early diastolic right ventricular collapse, consistent with cardiac tamponade, and emergency pericardial drainage was performed. Cytological examination of pericardial fluid revealed atypical cells, suggesting pericardial metastasis. As the left pleural effusion increased and required drainage, pleural fluid cytology revealed atypical cells. CT demonstrated an atelectasis-like mass along the mediastinal aspect of the left upper lobe, corresponding to the FDG-avid lesion on PET-CT. Additional findings suggested mediastinal invasion and disease progression, including right hilar lymphadenopathy, increased left pleural effusion, and left lung–predominant interlobular septal and peribronchovascular thickening, suggestive of pulmonary lymphangitic carcinomatosis. Whole-body bone scintigraphy demonstrated diffuse abnormal radiotracer uptake with a symmetric, periosteal-predominant distribution, characteristic of HPOA (12). Intense uptake was noted in the sacroiliac joints and sternum, with nodular uptake in the posterior and lateral ribs, suggesting active periostitis; mildly increased uptake was present in the tibial cortex and ankle joints (**Figure 3A**). Hand radiographs showed increased soft-tissue density, consistent with digital clubbing (**Figure 3B**), and MRI of the ankles demonstrated high periosteal signal intensity on fat-suppressed T2WI, supporting periostitis (**Figure 3C**). Collectively, HPOA was diagnosed.

In early September 20XX, the patient’s ECOG performance status (PS) was 2, primarily due to arthralgia and dyspnea. Given the rarity of EHE and the complexity of the clinical presentation, her diagnosis and treatment strategy were reviewed at a multidisciplinary tumor board, which included medical oncologists, surgical oncologists, radiation oncologists, diagnostic radiologists, and pathologists. Systemic therapy with paclitaxel (175 mg/m²), carboplatin (AUC 6), and bevacizumab (15 mg/kg) (TC+Bev) was initiated for symptom palliation and prolongation of survival. Zoledronic acid (4 mg) was also administered for the management of HPOA. After the first cycle, she developed grade 2 nausea (CTCAE v5.0) and transient arthralgia grade 2 due to paclitaxel, which were manageable. After three cycles of TC+Bev, follow-up CT demonstrated radiographic regression with marked resolution of pericardial and pleural effusions. Arthralgia improved, and her ECOG PS recovered to 1, reflecting meaningful improvement in activities of daily living.

However, in late November 20XX, arthralgia, dyspnea, and cough worsened. In early December 20XX, CT demonstrated radiographic progression, with an enlarging infiltrative lesion in the left upper lobe and increased left pleural effusion, resulting in near-complete opacification of the left lung. Concomitantly, new reticular opacities and interlobular septal thickening emerged in the right lower lobe, consistent with progression of pulmonary lymphangitic carcinomatosis. Pazopanib was started at 800 mg/day but was discontinued after two weeks because of the lack of clinical benefit and deterioration in performance status (ECOG PS 3). After rapid clinical deterioration, she was transitioned to palliative care and died in late December 20XX.

## 3 Discussion

This case report highlights two clinically important aspects of advanced PEH in a rare and life-threatening setting characterized by cardiac tamponade, massive pleural effusion, and HPOA. First, systemic chemotherapy in combination with bevacizumab was associated with transient radiographic and symptomatic improvement, including temporary control of pericardial and pleural effusions, despite subsequent rapid progression. Second, arthralgia and functional status improved during treatment with systemic chemotherapy and zoledronic acid, in parallel with temporary tumor control.

The prognosis of EHE is influenced by the tumor size at diagnosis, presence of metastasis, pleural or peritoneal effusions, and systemic symptoms. Pleural involvement in effusions has been associated with poor outcomes in previous studies (1, 2). Pericardial involvement with effusion is rare (14), and only a few cases complicated by cardiac tamponade have been reported (15, 16), most of which showed poor outcomes, underscoring the importance of prompt pericardial drainage and systemic disease control.

Although no standard systemic therapy has been established for EHE, the biological characteristics of EHE offer a therapeutic rationale. Considering the strong association between EHE and VEGF pathway activation, anti-angiogenic agents and molecular targeted therapies have been explored in EHE.

Bevacizumab has been evaluated in a phase II study in EHE (17), and in other malignancies, it is known to reduce pleural and pericardial effusions by neutralizing VEGF and inhibiting vascular hyperpermeability (18–20). In the present case, TC+Bev was associated with radiographic regression of the mediastinal lesion and transient improvement in pericardial and pleural effusions. Although reports of bevacizumab-containing regimens for PEH are limited, this case illustrates a distinct clinical course of effusion-dominant PEH, in which anti-VEGF therapy was associated with rapid but transient control of life-threatening serosal effusions, suggesting that anti-VEGF therapy may represent a potential treatment option for patients with PEH presenting with cardiac tamponade and pleural effusion.

After rapid disease progression, other anti-angiogenic and molecular targeted therapies were considered as subsequent treatment options. Sorafenib has been evaluated in a prospective phase II study in EHE, suggesting potential efficacy in disease stabilization (21); however, it has not been evaluated in a phase III trial for EHE and is not approved for soft tissue sarcoma in Japan or the United States. Pazopanib, by contrast, is approved for advanced soft tissue sarcoma based on the phase III PALETTE trial (22), although evidence supporting its use in EHE remains limited to retrospective studies and case reports (23–25). In the present case, pazopanib was therefore selected as a subsequent systemic treatment option; however, it was discontinued after two weeks because of the lack of clinical benefit and deterioration in performance status.

Here, digital clubbing, periosteal reaction, and joint swelling were observed, and HPOA was diagnosed based on clinical and imaging findings. The proposed mechanisms of HPOA include platelet–endothelial interactions, elevated von Willebrand factor (vWF), and periosteal new bone formation driven by VEGF overexpression (12). Few cases of HPOA are associated with EHE (6–11). No disease-specific treatment has been established for HPOA, and control of the underlying malignancy is the most important therapeutic goal. Although evidence is limited to case reports, bisphosphonates have been reported to provide rapid pain relief in HPOA refractory to NSAIDs and corticosteroids (13). However, evidence supporting the use of anti-VEGF therapy in HPOA remains scarce. Because VEGF overexpression may contribute to its pathogenesis, anti-VEGF therapy could ameliorate its symptoms; however, clinical evidence remains limited. In the present case, arthralgia improved after zoledronic acid administration. Tumor regression achieved with TC+Bev may have contributed to improved HPOA. As multiple agents were administered, the relative contribution of each drug could not be determined; however, a combined therapeutic effect is plausible.

## 4 Conclusion

In summary, we report an early-onset case of PEH complicated by HPOA and cardiac tamponade. Treatment with TC+Bev plus bisphosphonates is associated with radiographic improvement and symptomatic relief. However, the duration of this benefit was limited, highlighting the need for more effective systemic therapies for PEH. Although causality cannot be inferred from a single case, these observations are consistent with the possibility that VEGF pathway activity contributes to disease behavior, serosal effusion formation, and may contribute to the development of HPOA in a subset of PEH. Accordingly, anti-VEGF-containing therapy may be considered a palliative systemic option in selected patients, although further validation using integrated molecular profiling is warranted.

## Data Availability

The original contributions presented in the study are included in the article/supplementary material. Further inquiries can be directed to the corresponding author.
